# Relationship between the place of living and mortality in patients with advanced heart failure

**DOI:** 10.1186/s12875-020-01213-x

**Published:** 2020-07-14

**Authors:** Miguel-Angel Muñoz, Raquel Garcia, Elena Navas, Julio Duran, José-Luis Del Val-Garcia, José-Maria Verdú-Rotellar

**Affiliations:** 1 Institut Català de la Salut, Barcelona, Spain; 2 Fundació Institut Universitari per a la recerca a l’Atenció Primària de Salut Jordi Gol i Gurina (IDIAPJGol), Carrer Sardenya 375, Entresol, 08025 Barcelona, Spain; 3 Department of Pediatrics, Obstetrics and Gynecology and Preventive Medicine, Universitat Autònoma de Barcelona, Bellaterra, Spain; 4 Clínica Sant Antoni. Institut Mèdic i de Rehabilitació, Barcelona, Spain; 5 Department of Medicine, Universitat Autònoma de Barcelona, Bellaterra, Spain

**Keywords:** Advanced heart failure, Rural health, Community healthcare, Health inequalities

## Abstract

**Background:**

Social and environmental factors in advanced heart failure (HF) patients may be crucial to cope with the end stages of the disease. This study analyzes health inequalities and mortality according to place of residence (rural vs urban) in HF patients at advanced stages of the disease.

**Methods:**

Population-based cohort study including 1148 adult patients with HF attended in 279 primary care centers. Patients were followed for at least 1 year after reaching New York Heart Association IV functional class, between 2010 and 2014.

Data came from primary care electronic medical records. Cox regression models were applied to determine the hazard ratios (HR) of mortality.

**Results:**

Mean age was 81.6 (SD 8.9) years, and 62% were women. Patients in rural areas were older, particularly women aged > 74 years (*p* = 0.036), and presented lower comorbidity. Mortality percentages were 59 and 51% among rural and urban patients, respectively (*p* = 0.030). Urban patients living in the most socio-economically deprived neighborhoods presented the highest rate of health service utilization, particularly with primary care nurses (p-trend < 0.001). Multivariate analyses confirmed that men (HR 1.60, 95% confidence interval (CI) 1.34–1.90), older patients (HR 1.05, 95% CI 1.04–1.06), Charlson comorbidity index (HR 1.16, 95% CI 1.11–1.22), and residing in rural areas (HR 1.35, 95% CI 1.09 to 1.67) was associated with higher mortality risk.

**Conclusions:**

Living in rural areas determines an increased risk of mortality in patients at final stages of heart failure.

## Background

According to data published by the World Bank, the percentage of rural populations fluctuates from 19 to 59% depending on the degree of development of the countries in question [1]. In spite of these figures, however, differences in health and disease patterns between rural and urban areas have not been widely studied [2].

Evidence concerning inequalities in health and mortality between rural and urban settings, has reported conflicting results with respect to the source and characteristics of the population [3, 4].

With respect to cardiovascular diseases, coronary heart disease mortality has been observed to be more prevalent in rural areas [5], and similar results have been published for heart failure (HF) although only regarding men [6].

Whilst the HF incidence rate has declined in recent years, its prevalence has increased, suggesting that survival over time is longer, probably due to better care and treatment [7]. Nevertheless, this improvement in survival is modest [8], and research in HF is considered one of the most important priorities [9]. Patients classified as New York Heart Association (NYHA) functional class III/IV present almost four-fold greater rates of mortality, and up to 58.3% of NYHA functional class IV dies after a five-year follow-up [10, 11]. However, due to its irregular evolution, HF is not usually considered a terminal disease, whilst at advanced stages it could be compared to malignant neoplasms [12].

Although many predictors of mortality among HF patients have been well identified [13], social and environmental determinants are not usually included in predictive models, particularly in patients at terminal stages of the disease. Some studies have shown worse prognoses regarding socio-economic position [14], social risk [15], health literacy [16] and urban areas of residence [17].

Even though gaps in the availability of general palliative care in rural areas have been reported [18], information about the differences in the lifetime of HF patients or health services utilization depending on their residence and socio economic level is scarce.

This study was aimed at analyzing health inequalities and mortality according to place of residence (rural vs urban) in HF patients at advanced stages of the disease (NYHA functional class IV) attended in the community by analyzing real world data.

## Methods

We followed a population-based cohort of adult patients presenting the most advanced stages of heart failure (NYHA functional class IV) between January 1st, 2010, and December 31st, 2014.

The inclusion date was taken to be when patients were first registered as having NYHA functional class IV in their primary healthcare electronic medical records during the study period. NYHA functional class IV was considered when patients diagnosed from HF were unable to carry on any physical activity without symptoms of HF, or symptoms of HF at rest [19]. The whole cohort was followed-up for at least 1 year from the inclusion date or until a fatal occurrence took place during the study period.

Information was collected from the primary care electronic medical records, through the Information System for the Development of Research in Primary Care (SIDIAP). This database contains data from 5.8 million individuals attended in 279 primary healthcare centers which attend 80% of the whole population of Catalonia (north-east Spain), and it has already been validated for use in cardiovascular research [20].

The database incorporates both administrative and clinical data which are encrypted to guarantee the confidentiality and anonymization of the information gathered for research purposes and provides data about diagnoses, clinical characteristics, comorbidity, laboratory and diagnostic tests, social and demographic variables, and performance in activities of daily living, tests to evaluate functional physical and mental status, drug prescriptions, and primary health care service utilization. Information regarding the patients’ vital status is also included and comes from the Central Insurance Register (RCA) (ie, if patient is death or alive).

Patients with HF were identified using the International Classification of Diseases, Tenth Revision (ICD 10), claim code I50.

The ICD 10 codes selected to register comorbidities were: diabetes (E10-E14), hypertension (I10-I15), coronary heart disease (I20-I25), stroke (I63-I65), atrial fibrillation (I48), chronic kidney disease (N18), chronic lower respiratory diseases (J40-J44), and cancer (C00-C97).

Clinical variables, laboratory analyses, and tests assessing functional were obtained from the patient’s consultation closest to the inclusion date, and missing values were imputed.

The Barthel index, which has proved helpful in assessing the functional status of a patient [21], and the Charlson index, used to predict ten-year mortality and healthcare resource utilization in patients with a range of comorbid conditions [22] were regularly collected during the nurses’ consultations.

To determine socio-economic status among the urban patients we employed the MEDEA Index which is an aggregated socioeconomic deprivation model which classify the population living in small geographical areas, according to the percentage of unemployment, manual and temporary workers, and individuals with insufficient education (less than primary school). The lowest quintile (Urban areas 1) represents individuals with the most favorable socio-economic position, and the upper one the worst (Urban areas 1). The unit of aggregation was the census tracts, which is the smallest territorial unit for which population data are available in our country. This index has been proven valid for urban areas although in rural ones it does not discriminate accurately [23].

Family networks, as well as living conditions, were assessed through an interview by a social workers or nurses using the Gijon social-family scale, which includes questions about housing conditions, family and social relationships and income [24].

Family network was explored by the following items: Living with relatives (physically independent), living with relatives (physically dependent), living with a partner, living alone (offspring close to their home), and living alone (offspring far from their home). We grouped the last two variables and classified family network as living alone or not.

Regarding housing conditions, we considered them inadequate when the house had structural barriers, humidity or incomplete facilities, no elevator or telephone or patients were living in a slum.

Health service utilization was computed as the number of consultations made with the family physician, primary care nurse, or specific primary care emergency services.

Rural residence was defined when patients lived in an area with less than 10,000 inhabitants, or the density of population was lower than 150 inhabit/km2, according to the Catalan Healthcare Administration classification [25].

The prescription of angiotensin converting enzyme inhibitors, angiotensin II receptor blockers, beta blockers, mineral corticoid antagonists, and loop diuretics was also collected.

The primary outcome was all-cause mortality occurring during the follow-up.

### Statistical analysis

Continuous variables were described by means, Standard deviation, and median, and percentages were used to describe categorical variables.

Variables included in the analyses were: sociodemographic, clinical and laboratory test data, comorbidity, consultations made to primary healthcare professionals, activities of daily living, presence of family networks, housing conditions and medication related to HF.

Living in urban or rural residence was the main independent variable and global mortality was the end point of the study.

The Anova test was performed to compare the number of visits according to the different levels of MEDEA deprivation index as a categorical variable.

Characteristics related to mortality and differences between urban and rural patients were first compared using chi-square test or Student t test.

Odds ratios were estimated by logistic regression models to evaluate the relationship between urban and rural residence and to evaluate the association between these characteristics, the place of residence and mortality.

Multiple imputation models were performed to manage the missing data by using the “Mice” and “VIM” R packages. The “mice” function is based on the conditional specification, where each incomplete variable is imputed with a separate model. A total of 50 imputations were generated and the final data were obtained with the “complete” function that generates a complete data set that combines the observed and imputed values.

Cox multivariate logistic regression was used to analyse the characteristics related to mortality and covariates included in the model. Socio demographic variables and those statistically significant (at level *p* < 0.01) in the bivariate analyses (sex, age, Charlson Index, place of residence, diabetes mellitus, and number of visits to primary care urgencies) were included in the final model.

Statistical analysis was conducted using R Software for Windows version 3.6.1, Vienna, Austria.

## Results

### Descriptive findings

We analyzed data from 1148 HF patients in NYHA functional class IV. Mean age was 81.6 (SD 8.9), 95% of patients were older than 64 years, and 61% were women. Most of the population lived in urban areas (*N* = 972) and more than 69% were older than 74 years (*N* = 793). Among the total number of patients included, 68% presented high comorbidity according to the Charlson index (score > =3), and daily living activities were moderately to severely affected (Barthel Index < 60) in 56%.

The most frequently associated comorbidities were hypertension (79%), atrial fibrillation (47%), diabetes (42%), and coronary heart disease (34%). Cardiovascular comorbidity (coronary heart disease, atrial fibrillation, and stroke) was present in 69% of the patients, and 22% presented two or more of these conditions.

Regarding the number of chronic conditions included in the analyses, the median was 3 (percentile 25–75, 2–4), and 62% of HF patients had simultaneously three or more comorbidities.

Beta-blockers, angiotensin converting enzyme inhibitors/angiotensin II receptor blockers, and mineral corticoid antagonists were prescribed in 45, 66, and 26% of patients, respectively. A combination of the three medications was present in 10%, whilst loop diuretics were prescribed in 88%.

During the period of the study, patients consulted their family physician on average 22 occasions, 20 with their primary care nurse, and on three occasions the primary healthcare emergency centers.

### Differences between urban and rural patients

Mean follow-up until the end of the study, or the occurrence of a fatal event, was 16 months (SD 12.4) for urban and 14.5 months (SD 10.9) for rural patients, respectively.

Although rural patients were, on average, 2 years older, they presented lower comorbidity (Charlson index). In spite of not being statistically significant, the urban cohort tended to live more commonly alone.

No differences in cardiovascular comorbidity, clinical variables, performance in activities of daily living, or HF medication were reported between rural and urban patients.

Table 1 describes how in analyses stratified by gender there were no outstanding differences regarding place of residence in any of the variables, with the exception of age (older women in rural areas) and the higher percentage of women living alone in the cities.

**Table 1:** Characteristics of patients with heart failure at New York Heart Association IV functional class, according to their place of residence

	Overall			Women			Men		
	Urban ***N*** = 972	Rural ***N*** = 176	***P*** value	Urban ***N*** = 598	Rural ***N*** = 110	***P*** value	Urban ***N*** = 374	Rural ***N*** = 66	***P*** value
Age*	81.3 (8.8)	83.0 (8.6)	0.020	82.9 (8.5)	84.5 (7.6)	0.039	79.0 (8.95)	80.5 (9.5)	0.228
Mortality	488 (51)	104 (59)	0.037	277 (46)	60 (54)	0.120	211 (56)	44 (67)	0.137
**Cardiovascular Risk**									
Body Mass Index^a^	30.4 (6.8)	29.4 (5.8)	0.112	31.6 (7.1)	30.5 (6.3)	0.186	28.7 (5.9)	27.6 (4.6)	0.154
Smoker	36 (4)	5 (3)	0.404	8 (1)	0 (0)	0.490	28 (7)	5 (8)	0.596
**Comorbidity**									
Diabetes	422 (43)	63 (36)	0.062	248 (42)	38 (35)	0.210	174 (47)	25 (38)	0.243
Coronary heart disease	326 (34)	49 (28)	0.163	162 (27)	22 (20)	0.150	164 (44)	27 (41)	0.757
Stroke	141 (15)	22 (13)	0.465	79 (13)	12 (11)	0.612	62 (17)	10 (15)	0.914
Chronic kidney disease	331 (34)	60 (34)	0.999	217 (36)	36 (33)	0.543	114 (31)	24 (36)	0.420
Chronic obstructive pulmonary disease	322 (33)	56 (32)	0.738	121 (20)	24 (22)	0.803	201 (54)	32 (49)	0.512
Atrial fibrillation	453 (47)	83 (48)	0.897	305 (51)	51 (46)	0.429	148 (40)	32 (49)	0.222
Cancer	183 (19)	33 (19)	0.989	96 (16)	18 (16)	1.000	87 (23)	15 (23)	1.000
Hypertension	774 (80)	135 (77)	0.384	500 (83)	94 (86)	0.732	274 (73)	41 (62)	0.089
**Clinical Variables**									
Heart rate (beats/minute) ^a^	77.0 (14.4)	76.0 (15.8)	0.456	76.8 (14.6)	75.5 (16.4)	0.450	77.1 (14.1)	76.8 (15.0)	0.895
Systolic blood pressure (mmHg)^a^	126.0 (18.6)	124.0 (18.4)	0.208	127.0 (19.0)	125.0 (18.1)	0.222	123.0 (17.9)	122.0 (19.2)	0.598
Diastolic blood pressure (mmHg)^a^	68.2 (10.6)	67.1 (10.6)	0.192	68.9 (11.0)	67.8 (9.8)	0.297	67.3 (9.9)	66.1 (11.9)	0.437
Potassium mEq/L ^a^	4.6 (0.6)	4.5 (0.5)	0.780	4.5 (0.5)	4.5 (0.5)	0.711	4.6 (0.6)	4.5 (0.5)	0.402
Sodium mEq/L ^a^	141.0 (3.7)	140.0 (3.9)	0.485	141.0 (3.6)	141.0 (3.8)	0.713	140.0 (3.8)	140 (4.0)	0.534
Glomerular filtration	46.7 (14.6)	46.6 (13.8)	0.894	44.7 (13.8)	44.3 (13.5)	0.796	49.9 (15.3)	50.3 (13.5)	0.853
Haemoglobin (g/dL)^a^	12.1 (1.8)	12.1 (1.7)	0.993	11.9 (1.6)	12.1 (1.6)	0.268	12.5 (1.9)	12.3 (1.9)	0.287
Charlson Index^a^	3.6 (1.8)	3.3 (1.5)	0.031	3.4 (1.7)	3.1 (1.4)	0.058	3.9 (1.9)	3.6 (1.6)	0.194
Barthel Index^a^	57.3 (27.9)	55.9 (31.1)	0.535	52.5 (27.3)	51.4 (29.6)	0.707	64.9 (27.2)	63.3 (32.5)	0.701
**Medication**									
Beta-blockers	448 (46)	69 (39)	0.098	279 (47)	46 (42)	0.406	169 (45)	23 (35)	0.154
ACEi or ARB ^b^	639 (66)	119 (68)	0.692	400 (67)	78 (71)	0.474	239 (64)	41 (62)	0.890
MRA ^c^	263 (27)	41 (23)	0.343	143 (24)	19 (17)	0.161	120 (32)	22 (33)	0.954
Loop diuretics	859 (88)	156 (89)	0.929	522 (87)	99 (90)	0.524	337 (90)	57 (86)	0.485
**Social Variables**									
Living alone	301 (31)	42 (24)	0.071	195 (33)	26 (24)	0.079	106 (28)	16 (24)	0.591
Inadequate housing conditions	424 (44)	83 (47)	0.384	243 (41)	54 (49)	0.122	181 (48)	29 (44)	0.593

^a^Mean (Standard deviation); ^b^*ACEi* angiotensin converting enzyme inhibitors and *ARB* Angiotensin II receptor blockers),^c^*MRA* mineralocorticoid receptor antagonists

**Fig. 1:**
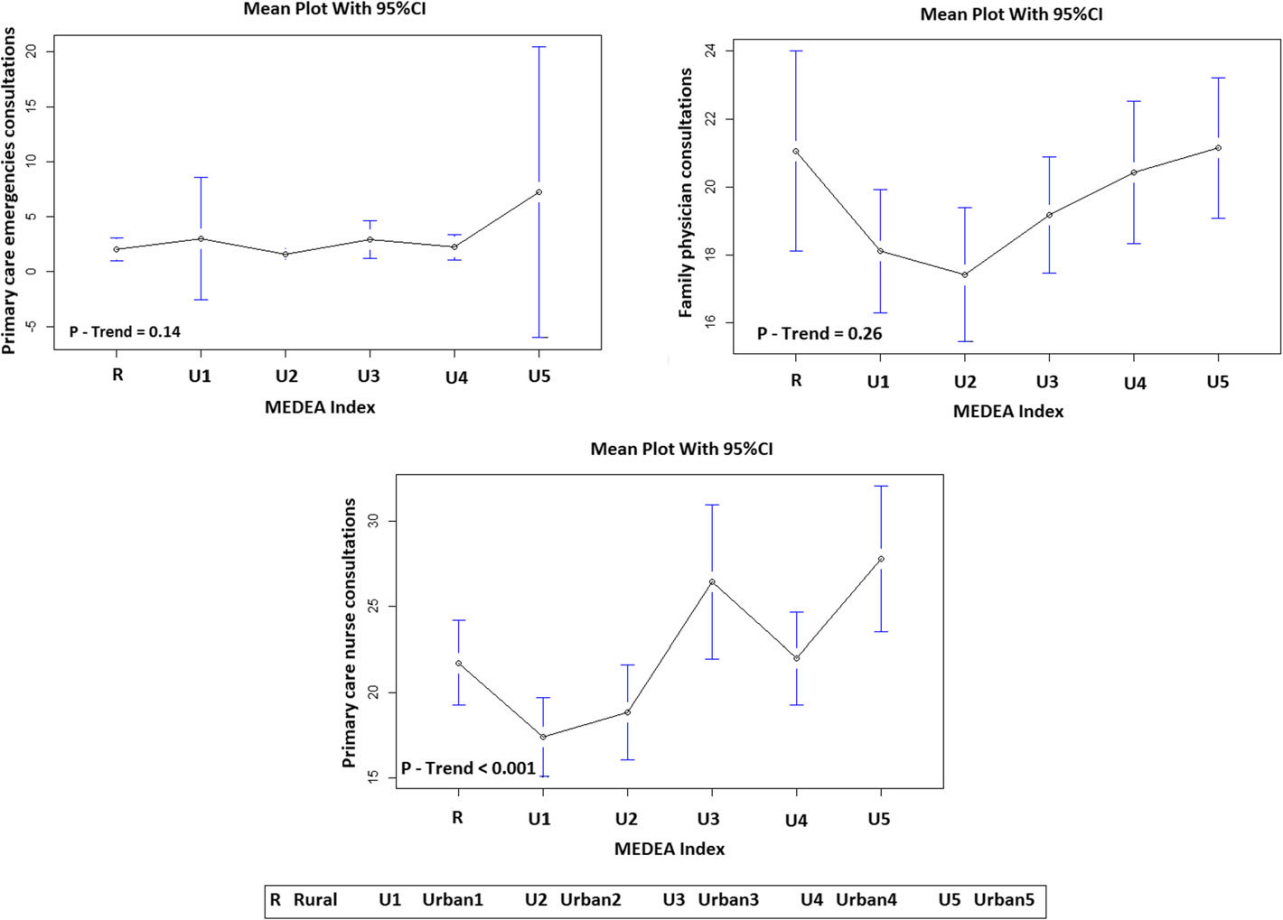
Primary healthcare services utilization by urban patients with heart failure at New York heart Association (NYHA) functional class IV according to the place of residence (U 1: urban less socioeconomically deprived area to U 5: urban most socioeconomically deprived area)

We compared primary healthcare service utilization by HF patients, according to place of residence and socioeconomic deprivation it was observed that those in the most socio-economically deprived urban areas tended to use more frequently primary care emergency services than the other urban individuals, and even three more times compared to rural ones. Regarding consultations with the family physician and primary healthcare nurses, patients in the most deprived urban areas were the greatest users of the former and this higher utilization was statistically significant in the case of the later (Fig. 1).

### Overall mortality

Among the 592 patients (52%) who died during the period of the study, 488 (82%) lived in urban and 104 (18%) in rural areas, respectively. Analyzing the number of fatal events during follow-up, the mean rate of survival from study inclusion was 10.6 (SD 9.9) and 9.8 (SD 9.8) months, for the urban and rural cohorts, respectively.

Older age, Charlson comorbidity index, chronic kidney disease, higher potassium levels, and cancer, were related with a higher risk of dying.

In contrast, greater body mass index, blood pressure, glomerular filtration, Barthel index, and hemoglobin were protective with respect to mortality risk (Table 2).

**Table 2:** Characteristics related to the overall mortality of patients with heart failure at New York heart Association IV functional class, according to gender

	Overall			Women			Men		
	Alive ***N*** = 556	Dead ***N*** = 592	***P*** value	Alive ***N*** = 371	Dead ***N*** = 337	***P*** value	Alive ***N*** = 185	Dead ***N*** = 255	***P*** value
Age	79.7 (9.3)	83.3 (8.0)	< 0.001	81.4 (8.6)	85.0 (7.7)	< 0.001	76.5 (9.9)	81.2 (7.7)	< 0.001
Body Mass Index	31.5 (6.7)	28.9 (6.4)	< 0.001	32.4 (6.8)	30.1 (7.0)	< 0.001	30.0 (6.2)	27.5 (5.2)	< 0.001
Smoker	22 (4)	19 (3)	0.698	7 (2)	1 (0)	0.112	15 (8)	18 (7)	0.009
**Comorbidity**									
Diabetes	234 (42)	251 (42)	0.971	154 (41)	132 (39)	0.577	80 (43)	119 (47)	0.538
Coronary heart disease	178 (32)	197 (33)	0.782	100 (27)	84 (25)	0.597	78 (42)	113 (44)	0.725
Stroke	78 (14)	85 (14)	0.854	50 (14)	41 (12)	0.683	28 (15)	44 (17)	0.644
Chronic kidney disease	162 (29)	229 (39)	< 0.001	119 (32)	134 (40)	0.040	43 (23)	95 (37)	0.003
Chronic obstructive pulmonary disease	170 (31)	208 (35)	0.089	76 (21)	69 (21)	1.000	94 (51)	139 (55)	0.502
Atrial fibrillation	254 (46)	282 (48)	0.707	184 (50)	172 (51)	0.758	70 (38)	110 (43)	0.309
Cancer	81 (15)	135 (23)	< 0.001	54 (15)	60 (18)	0.284	27 (15)	75 (29)	< 0.001
Hypertension	449 (81)	460 (77)	0.218	313 (84)	281 (83)	0.800	136 (74)	179 (70)	0.513
**Clinical Variables**									
Heart Rate (beats/minute) ^a^	76.6 (15.2)	76.8 (14.0)	0.735	76.7 (15.4)	76.5 (14.3)	0.798	76.4 (14.9)	77.4 (13.7)	0.487
Systolic blood pressure (mmHg)^a^	127.7 (18.4)	123.3 (18.5)	< 0.001	129 (18.3)	125 (19.2)	0.007	126 (18.4)	121 (17.5)	0.009
Diastolic blood pressure (mmHg)^a^	69.5 (10.7)	66.7 (10.3)	< 0.001	70.2 (11.1)	67.1 (10.3)	< 0.001	68.2 (9.8)	66.3 (10.4)	0.051
Potassium mEq/L ^a^	4.5 (0.5)	4.6 (0.6)	0.033	4.4 (0.5)	4.5 (0.5)	0.133	4.5 (0.6)	4.6 (0.6)	0.370
Sodium mEq/L ^a^	140.8 (3.5)	140.5 (3.9)	0.060	141 (3.4)	141 (3.7)	0.351	141 (3.5)	140 (3.9)	0.309
Glomerular filtration	1.2 (0.6)	1.4 (0.8)	< 0.001	1.1 (0.4)	1.3 (0.5)	< 0.001	1.3 (0.8)	1.5 (1.0)	0.007
Hemoglobin (g/dL)^a^	12.3 (1.7)	11.9 (1.8)	< 0.001	12.2 (1.5)	11.7 (1.7)	< 0.001	12.8 (1.9)	12.3 (1.9)	0.009
Barthel Index ^a^	59.8 (27.8)	54.4 (28.6)	< 0.001	55.9 (27.2)	48.5 (27.6)	< 0.001	67.8 (27.5)	62.4 (28.2)	0.043
Charlson Index ^a^	3.2 (1.6)	3.8 (1.8)	< 0.001	3.2 (1.5)	3.5 (1.6)	0.024	3.4 (1.6)	4.2 (1.9)	< 0.001
**Medication**									
Beta-blockers	271 (49)	246 (42)	0.011	182 (49)	143 (42)	0.091	89 (48)	103 (40)	0.130
ACEi or ARB ^b^	397 (71)	361 (61)	< 0.001	262 (71)	216 (64)	0.077	135 (73)	145 (56)	0.001
MRA^c^	145 (26)	159 (27)	0.883	83 (22)	79 (23)	0.803	62 (33)	80 (31)	0.711
Loop diuretics	479 (86)	536 (91)	0.083	315 (85)	306 (91)	0.023	164 (89)	230 (90)	0.714
**Social Variables**									
Living alone	164 (29)	179 (30)	0.988	121 (33)	100 (30)	0.446	43 (23)	79 (31)	0.093
Inadequate housing conditions	220 (40)	287 (48)	0.002	131 (35)	166 (49)	< 0.001	89 (48)	121 (47)	0.968

^a^Mean (Standard deviation); ^b^*ACEi* angiotensin converting enzyme inhibitors, *ARB* Angiotensin II receptor blockers);^c^*MRA* mineralocorticoid receptor antagonists

When analyzing sociodemographic variables related to mortality we found that being men, older, living in rural areas and having unfavorable housing conditions (structural barriers and lack of facilities) were related to higher probability of dying, but no significant differences were found among urban patients regarding the area where they lived.

Table 3 shows the differences in mortality according to the fact of living in urban and rural areas. Older age, chronic kidney disease, lower levels of blood pressure, lower levels of haemoglobine and lower body mass index were more commonly related to mortality, regardless the place of residence. We observed that in patients living in rural areas the prevalence of stroke among those patients who died was higher, whilst in urban patients mortality was higher in those with higher prevalence of cancer, lower Barthel Index and worse levels of creatinine.

**Table 3:** Characteristics related to the mortality of patients with heart failure at New York Heart Association IV functional class according to their place of residence

	Overall			Urban			Rural		
	Alive ***N*** = 556	Dead ***N*** = 592	***P*** value	Alive ***N*** = 484	Dead ***N*** = 488	***P*** value	Alive ***N*** = 72	Dead ***N*** = 104	***P*** value
Age	79.7 (9.3)	83.3 (8.0)	< 0.001	79.7 (9.5)	83.0 (7.9)	< 0.001	79.9 (8.5)	85.1 (8.0)	< 0.001
Body Mass Index ^a^	31.5 (6.7)	28.9 (6.4)	< 0.001	31.7 (6.8)	29.1 (6.5)	< 0.001	31.0 (5.7)	28.0 (5.6)	0.004
Smoker	22 (4)	19 (3)	0.698	20 (4)	16 (3)	0.532	2 (3)	3 (3)	0.801
**Comorbidity**									
Diabetes	234 (42)	251 (42)	0.971	204 (42)	218 (45)	0.466	30 (42)	33 (32)	0.233
Coronary heart disease	178 (32)	197 (33)	0.782	160 (33)	166 (34)	0.804	18 (25)	31 (30)	0.597
Stroke	78 (14)	85 (14)	0.854	74 (15)	67 (14)	0.549	4 (5)	18 (17)	0.037
Chronic kidney disease	162 (29)	229 (39)	< 0.001	146 (30)	185 (38)	0.013	16 (22)	44 (42)	0.009
Chronic Obstructive Pulmonary disease	170 (31)	208 (35)	0.089	150 (31)	172 (35)	0.180	20 (28)	36 (35)	0.428
Atrial fibrillation	254 (46)	282 (48)	0.707	224 (46)	229 (47)	0.891	30 (42)	53 (51)	0.289
Cancer	81 (15)	135 (23)	< 0.001	70 (15)	113 (23)	0.001	11 (15)	22 (21)	0.432
Hypertension	449 (81)	460 (77)	0.218	388 (80)	386 (79)	0.739	61 (85)	74 (71)	0.056
**Clinical Variables**									
Heart rate^a^	76.6 (15.2)	76.8 (14.0)	0.735	76.5 (14.7)	77.3 (14.2)	0.413	77.5 (18.8)	75.0 (13.4)	0.329
Systolic blood pressure (mm Hg)^a^	127.7 (18.4)	123.3 (18.5)	< 0.001	128 (18.3)	124 (18.9)	0.002	128 (19.5)	121 (17.1)	0.008
Diastolic blood pressure (mm Hg)^a^	69.5 (10.7)	66.7 (10.3)	< 0.001	69.6 (10.7)	67.0 (10.3)	< 0.001	69.2 (10.7)	65.7 (10.4)	0.031
Potassium (mEq/dL)^a^	4.5 (0.5)	4.6 (0.6)	0.033	4.5 (0.5)	4.5 (0.6)	0.082	4.5 (0.4)	4.6 (0.5)	0.246
Sodium (mEq/dL)^a^	140.8 (3.5)	140.5 (3.9)	0.060	141 (3.4)	141 (3.8)	0.180	141 (3.8)	140 (3.9)	0.455
Creatinine^a^	1.2 (0.6)	1.4 (0.8)	< 0.001	1.2 (0.6)	1.5 (0.8)	< 0.001	1.2 (0.5)	1.3 (0.6)	0.171
Haemoglobin (gr/dL)^a^	12.3 (1.7)	11.9 (1.8)	< 0.001	12.3 (1.8)	11.9 (1.8)	< 0.001	12.5 (1.5)	11.9 (1.8)	0.013
Barthel Index imputed^a^	59.8 (27.8)	54.4 (28.6)	< 0.001	59.7 (27.3)	54.9 (28.3)	0.007	60.8 (31.6)	52.5 (30.5)	0.083
Charlson Index^a^	3.2 (1.6)	3.8 (1.8)	< 0.001	3.3 (1.6)	3.8 (1.8)	< 0.001	2.9 (1.2)	3.5 (1.6)	0.005
**Medication**									
Beta-blockers	271 (49)	246 (42)	0.011	235 (49)	213 (44)	0.142	36 (50)	33 (32)	0.022
ACEi or ARB^b^	397 (71)	361 (61)	< 0.001	339 (70)	300 (62)	0.006	58 (81)	61 (59)	0.004
MRA^c^	145 (26)	159 (27)	0.883	130 (27)	133 (27)	0.947	15 (21)	26 (25)	0.644
Loop diuretics	479 (86)	536 (91)	0.083	416 (86)	443 (91)	0.025	63 (88)	93 (89)	0.878
**Social Variables**									
Living alone	164 (29)	179 (30)	0.988	146 (30)	155 (32)	0.639	18 (25)	24 (23)	0.909
Inadequate housing conditions	220 (40)	287 (48)	0.002	187 (39)	237 (49)	0.002	33 (46)	50 (48)	0.889

^a^Mean (Standard deviation); ^b^*ACEi* angiotensin converting enzyme inhibitors, *ARB* Angiotensin II a receptor blockers; ^c^*MRA* mineralocorticoid receptor antagonists

### Multivariate analyses

**Fig. 2:**
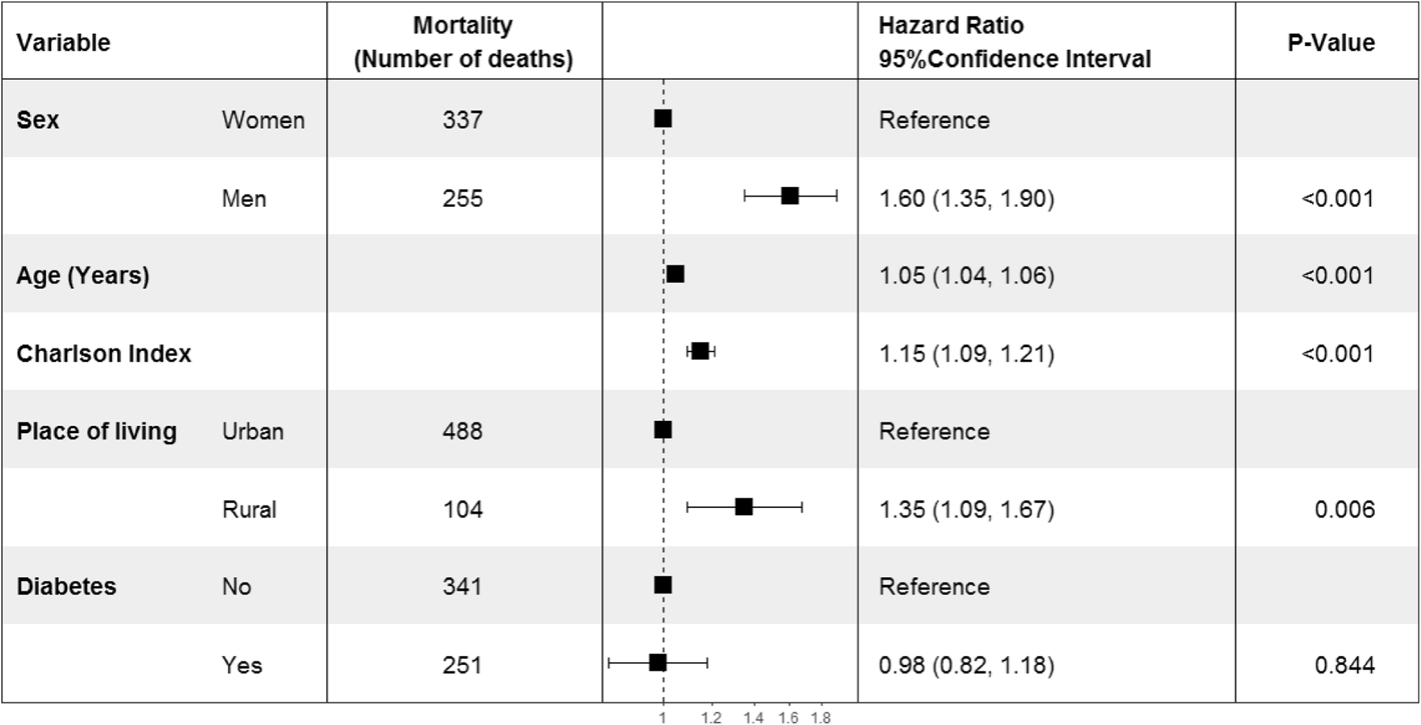
Hazard ratio and 95% confidence interval for variables predicting mortality adjusted by sex, age, Charlson comorbidity index, diabetes mellitus and place of living

Cox multivariate regression model, adjusted by the variables significantly associated both with the mortality and the place of living, confirmed that being men, older, having higher comorbidity, and living in rural areas were associated with higher risk of mortality (Fig. 2). The excess of mortality risk for men was 60% whilst for patients living in rural areas it was 35% (HR 1.35, 95% CI 1.09 to 1.67).

## Discussion

In our study, based on a community cohort of HF patients at the most advanced stages of the disease, we observed that mortality was 35% higher in those residing in rural setting. Being men, aged, and presenting higher comorbidity were also found to be related to a greater risk of dying. We found an inverse gradient in primary healthcare resources utilization regarding socio-economic deprivation among urban patients, those residing in the most deprived socio-economic areas had the highest health services utilization.

Compared to the rest of Europe, the population density in the study region is among one of the highest, the distribution is, however, irregular with more than 40% of the inhabitants concentrated in the metropolitan area of Barcelona. Although a number of small hospitals are distributed across the territory, most tertiary hospitals are located in this large metropolitan area which can limit accessibility of rural patients to highly specialized care.

With respect to place of residence, we did not observe differences in clinical characteristics or HF medication prescribed. Although rural women were slightly older, urban patients presented higher comorbidity and reported more social isolation. This last finding concurs with a previous study reporting that in a rural environment it is easier to rely on family members [26].

Although the bivariate analyses found statistical differences in the characteristics associated with mortality depending on rural or urban setting, these differences were not clinically relevant.

We found a considerable number of consultations, particularly with primary care nurses, among the urban patients living in the most deprived areas. In this regard, it has already been reported that low income and other psycho-social disadvantages imply greater healthcare resource utilization [27]^.^

### Mortality

The higher mortality described for men, older HF patients, and in those with decreased body mass index, chronic kidney disease, and lower blood pressure levels, concurs with previous studies, particularly in the advanced stages of the disease [28–30]. Moreover, higher mortality in rural patients is in agreement with a previous study [6].

It has been hypothesized that populations residing in urban areas have better health due to easier accessibility to health services, in addition to better jobs and income [3].

Regarding cardiovascular diseases, inequalities in rural patients have been reported with respect to access to treatment, such as percutaneous coronary interventions, which require a longer travelling distance [31].

Another possible factor contributing higher mortality among rural HF patients in our sample could be the differences reported in the use of health services, although we cannot affirm that highest primary care services utilization by urban patients determines longer survival.

There is a widespread network of primary healthcare out-of-hours centers, nevertheless, its distribution and characteristics vary. These centers are more complete in large cities, offering services such as radiology and laboratory tests, which may improve the diagnosis and treatment of complications whilst in the rural areas more complex assistance needs to be provided in small hospitals.

Many rural patients lack daily access to their family doctors which can lead to delay in the treatment of decompensations. Urban primary healthcare centers are opened between 8 am and 8 pm every day, whilst many rural villages do not have general practitioner accessible all days, and population must travel long distances to be attended, especially in out of hours.

Other factors might be involved in explaining in these differences such as the decision to die at home allows patients to maintain control over their lives. The approach to the end of life could be culturally different in a rural setting compared to an urban one. A recent systematic review oriented to analyse the socio-economic factors determining the place of death found that dying at home was associated with living in rural areas but, potential causes remain unsolved [32]. Mean survival time among patients who died in our study was slightly higher among the urban ones. Nevertheless, considering the considerable limitations such patients undergo in their daily activities, it may be questioned whether this increased survival time is cost effective in terms of quality of life.

Evidence regarding socioeconomic deprivation among urban residents is controversial. Hawkins et al., employing a geographical composite deprivation index similar to ours, and analyzing data from 2000 to 2007, did not describe differences regarding outcomes in HF patients [33]. In a more recent article, Witte et al., using the same deprivation index as Hawkins, reported that socioeconomic deprivation was linked to an increased risk of death in HF patients, but only as a consequence of non-cardiovascular causes [34]. In addition, a study performed with the same population in our country showed a protective, although not significant, effect regarding mortality in the most deprived urban patients [17].

The National Health Service in our country provides universal healthcare, which may reduce social inequalities in health by facilitating access to primary care, prescriptions and hospitals to populations lacking economic resources, but the distribution of healthcare premises in rural and remote areas is conditioned by geographical limitations.

Nevertheless, future research will be needed to explain why, with no differences in either cardiovascular comorbidities or treatment, rural HF patients had the highest mortality rates.

### Strengths and limitations

Although different approaches have been employed to define rurality, our definition concurs with others used in similar articles. Nevertheless, due to data limitations, we could not fully discriminate the analyses between patients living in the most isolated areas from the other rural ones to ascertain whether differences in accessibility related to mortality are proportional to distance and frequency of healthcare service provision. The deprivation index used to study urban socioeconomic differences assumes homogeneity among the population living in the same geographical area, but may imply an ecological fallacy because it is possible to find both poor and affluent individuals in the same areas, sometimes divided by only one street. Moreover, we lack information in order to discriminate the presence of socio-economic differences within rural populations and thus considered them homogeneous in terms of social status.

Since administrative databases are used for clinical purposes can lead to missing data. In the case of HF some variables such as ejection fraction are not always available to have proper diagnoses according to guidelines [35]. Nevertheless in our study this fact is not relevant because all patients were at final stages of the disease. Regarding other possible missing values we performed multiple imputation models to minimize such an effect.

It would be advantageous to possess data regarding quality of life in order to analyze differences among rural and urban patients.

The results of our study are potentially valid for areas of the rest of our country with similar characteristics, concentrating population in large cities. Nevertheless, in Spain and in Europe there are many areas where the rural depopulation is remarkable, and the accessibility to healthcare services could be different.

Since our results are based on information coming from more than 80% of the Catalan population, it is expected to think that they could be extrapolated to the rest.

Prospective studies would be needed, not just to observe the presence of differences depending on the place of living but oriented towards specifically analyse their causes, to be more precise in measuring the differences in the accessibility, the characteristics of patients, exploring their perceptions and needs as well as their beliefs and preferences regarding the treatment received at the end of life.

To our knowledge, evidence about differences between rural and urban heart failure patients, particularly at advances stages of the disease is scarce. This article specifically analyses evidence from electronic health records from elderly HF patients at advanced stages of the disease through a large database.

## Conclusions

Mortality in elderly patients with heart failure at the final stage of HF is higher among those living in rural setting. Accessibility and inequalities in the healthcare provided with respect to the place of residence may contribute to such differences. The increased healthcare services utilization by urban patients living in most socioeconomic deprived areas is not followed by a reduction in mortality.

Health policies should face with social and geographical inequalities, to ensure that most part of population have similar access to healthcare provision, especially to primary care services, which are essential in improving health and reducing mortality.

## Data Availability

The datasets generated and/or analysed during the current study are not publicly available due to the restrictions by the data owner (IDIAP Research Institute), but could be available from the corresponding author on reasonable request.

## Abbreviations

HF: Heart failure
NYHA: New York Heart Association
HR: Hazard ratios
CI: Confidence interval
RCA: Central Insurance Register
SIDIAP: Information System for the Development of Research in Primary Care.
